# Analyzing barriers and facilitators to the implementation of an action plan to strengthen the midwifery professional role: a Moroccan case study

**DOI:** 10.1186/s12913-015-1037-3

**Published:** 2015-09-15

**Authors:** Sabina Abou-Malham, Marie Hatem, Nicole Leduc

**Affiliations:** IRSPUM, Université de Montréal, P.O. Box 6128, Centre-ville Station, Montreal, QC H3C 3 J7 Canada; Department of Social and Preventive Medicine, School of Public Health, Université de Montréal, Montreal, Quebec Canada; Department of Health Administration, School of Public Health, Université de Montréal, Montreal, Quebec Canada

## Abstract

**Background:**

As part of a national strategy for reaching Millennium Development Goals 4 and 5 in Morocco, an action plan covering three systems (sociocultural, educational and professional) was developed to strengthen midwives’ professional role in order to contribute to high quality maternity care. This study aimed to understand the implementation process by identifying the characteristics of this intervention and the dimensions of the three-systems which could act as barriers to/facilitators of the implementation process. We used a conceptual framework that builds on Hatem-Asmar’s model that describes change in a health professional role; and on the Consolidated Framework for Implementation Research for our analysis.

**Methods:**

An embedded case study with three levels of analysis was conducted during June and July 2010. Data were collected through 11 semi-structured interviews, 20 focus groups, training session observations and documents. A purposive sample of 106 multi-stakeholders from two Moroccan regions (health professionals, academic staff, students, medical administrative officers and health programmers) and one international consultant were recruited. A thematic analysis was conducted using QDA Miner.

**Results:**

Data showed a failure to carry out the plan as intended. Seventeen barriers and seven facilitators were identified. Misalignment of the *values, methods, actors and targets* of the sociocultural system with the *values, methods and actors* of the educational and professional systems, on one hand, and with the intervention, on the other hand, were likely the greatest impediments to implementing the plan. The bureaucratic structure and lack of readiness of the sociocultural system were among the most influential barriers to: dissemination of information, involvement of key actors in the process and readiness of the educational system. The main facilitators were the values promoted related to human rights, and the national and international policies to strengthen midwifery and reduce maternal mortality. The plan was perceived as beneficial, but complex and externally driven.

**Conclusions:**

The findings suggest that successful implementation requires redesigning the implementation strategy to adapt to the factors identified in our study. The results would be very useful to health planners seeking the expansion of such an intervention to other developing countries looking to strengthen midwives’ role and to improve maternity health care services.

**Electronic supplementary material:**

The online version of this article (doi:10.1186/s12913-015-1037-3) contains supplementary material, which is available to authorized users.

## Background

Strengthening midwives’ professional role is a cornerstone in the process of improving the quality of maternal and newborn health care services and reducing maternal and neonatal mortality [1]. It is at the heart of many international strategic directions to support the achievement of Millennium Development Goals (MDGs) 4 (reduce infant mortality) and 5 (improve maternal health) [1–3]. Indeed, more than 80 % of maternal deaths, stillbirths, and neonatal deaths could be prevented by midwifery interventions targeting reproductive, maternal and newborn health [4]. To tackle this challenge, calls to actions have been made in African countries to implement skill development interventions for health professionals in general and for midwives in particular [2]. Role strengthening is one of the main interventions being considered to provide high-quality care through expanding human resources’ skills, tasks and responsibilities beyond their traditional scope of practice [5]. It has been also conceptualized as a whole systems change implicating essential actions in many systems, including the sociocultural, and educational systems as well as the system pertaining to the profession itself^1^ [6], through which a health professional role (namely the midwife’s role) evolves. However, it is well documented that a health workforce intervention is a multisystem intervention involving coordinated efforts at multiple levels and affecting different actors [7, 8]. Therefore, it is contingent upon the context in which it is implemented [5, 8] and runs the risk of not being implemented as intended, compromising the achievement of the intended goals [9]. Consequently, implementation challenges must be studied in order to fine-tune the intervention and to provide timely solutions for increasing the chances of producing desired effects [10, 11]. Indeed, international recommendations such as those of the State of the World Midwifery report in 2011 emphasize that scientific evaluations of interventions targeting the midwifery workforce should be conducted. To date, few evaluation and implementation studies have addressed midwifery interventions and, more specifically, interventions aiming to strengthen midwives’ professional role. A recent systematic review has reported that three main challenges were encountered when implementing task-shifting midwifery interventions in high, middle and low income countries and must be considered in the future to ensure a successful implementation [7]. They relate to: defending the midwifery model of care; training, supervision and support; teamwork and task shifting. The focus of this review was mainly the identification of implementation factors associated with task-shifting interventions covering various forms of vertical (e.g., from doctors to midwives) and horizontal (e.g., from midwives to nurses) shifts and shifts involving many types of human resources among whom tasks can be shifted. The challenges for extending midwives’ skills highlighted in this review mainly involved complex/illness-related tasks or a wider range of other tasks such as management of gestational diabetes mellitus, genetic and cervical cancer screening and abortion services. While identifying barriers and facilitators to implementing all forms of task-shifting seems useful, it does not explain specific issues, such as how to best to implement a particular type of skill development intervention, within the health workforce management strategy, to strengthen a professional role. With regards to deepening our understanding of the implementation process of interventions related to strengthening the midwifery role and, specifically, those adopting a systems approach^2^ adapted to the context in which the midwifery professional role evolves, the literature falls short. Further research is needed in this area to inform policy makers’ decisions and to tailor interventions to the factors that have been identified in the context under investigation [12]. To address this need, this paper presents findings from an implementation analysis of an action plan (AP) to strengthen midwives’ professional role in Morocco with the aim of reducing the scientific knowledge gap on role strengthening interventions. The objectives of this study are to: 1) assess the extent to which the intervention was delivered; and 2) understand how the possible barriers and facilitators influenced the AP’s implementation in Morocco.

### Intervention background

Morocco is recognized as one of the North-African countries that have made maternal health a top priority and exerted substantial efforts to achieve MDG 5. In 2008, the Ministry of Health (MOH) in Morocco launched a national action plan to reduce the maternal mortality ratio from 227 deaths per 100 000 live births to 50 per 100 000 by 2012. It included three main pillars: i) reducing barriers to access to emergency obstetric care and improving the availability of qualified personnel; ii) improving the quality of care during pregnancy and delivery; and, iii) improving governance and management [13]. A central component of the first pillar, was strengthening the competencies of health professionals, including midwifery competencies in terms of enhancing: i) midwifery pre-service education through reviewing the midwifery curriculum according to the competency-based approach (CBA); and, ii) in-service training by providing a continuing education program [14].

To fulfill this commitment, the MOH with the support of the United Nations Population Fund (UNFPA) collaborated with the Université de Montréal. An assessment of the state of midwifery in Morocco revealed that the profession faced substantial challenges related to education and other issues that need to be addressed. They pertained to: i) the lack of congruence of the curriculum with international standards; ii) a negative image of midwifery within the professional community and the society at large; iii) an old legal framework governing the profession resulting in an ill-defined scope of practice which is not built upon the ICM international definition and does not allow midwives to provide women-centered care; and, iv) the unfavorable practice environment [15]. In sum, it showed that midwives were trained three years at the undergraduate level with an underlying philosophy focusing on illness and a medical model rather than on wellness and comprehensive health care approach. The educational system prepared them to work as "technicians of birth" according to a biomedical model that shapes their practice, in a sociocultural system that does not value their status [15]. Following this assessment, a researcher from the Université de Montréal, developed an action plan (AP), aiming to strengthen the midwife’s professional role, consisting of many activities in addition to the educational activities initially prescribed by the MOH. Such a plan would enable midwives to fulfill their role in relation to the international definition of the midwife [16] in order to better meet the needs of Moroccan women and their families. It was designed in terms of the following objectives "see Additional file 1": i) educational objectives (e.g., developing and implementing a competency-based education program (CBE); ii) professional objectives (e.g., revitalizing the midwives association); iii) sociopolitical, legal objectives (e.g., setting-up marketing activities). The main objective of the AP was to provide qualified midwives functioning in an enabling environment in order to improve the quality of maternal and newborn health care delivery and outcomes (Hatem, M: Mission report Technical assistance for the revision of the midwifery education program in Morocco, unpublished).

### The conceptual framework

The framework [17] builds on Hatem-Asmar et al.’s conceptual model [6] that describes change in a health professional role and on Damschroder et al.’s Consolidated Framework for Implementation Research (CFIR) for implementation analysis [18]. Hatem-Asmar et al. consider three systems (*sociocultural, educational, professional*), including their dimensions (*values, methods, actors, targets*), to have an interactional relationship within which a health professional role operates; these should be considered as a whole system for addressing a health professional role change. Damschroder et al.’s CFIR provides a taxonomy of constructs, that are likely to influence implementation of complex interventions, organized into five domains: 1) Intervention characteristics; 2) Outer setting; 3) Inner setting; 4) Characteristics of individuals; and 5) Process of implementation. The Hatem-Asmar model served to identify the context in which the implementation process took place, referring to the three systems targeted by the intervention. Whereas, the CFIR’s "menu of constructs" was used as a framework to analyze and organize the findings related to the implementation process (Executing), to the influential factors at play in the three systems and within the intervention by inductively mapping themes onto the CFIR constructs. Constructs of two domains (Inner and Outer settings) were applied to the dimensions of the three systems as in our case there is no single set of Inner/Outer settings due to the complex nature of the interrelated systems under study. The characteristic of our framework is that it considers a set of dimensions in each system, arguing that barriers or facilitators identified according to the CFIR’S "menu of constructs" could exist in these dimensions interacting with the intervention which should be examined in order to understand the implementation process.

## Methods

### Study design

A case study design was chosen to conduct an implementation evaluation. The *case* was the midwifery profession undergoing the implementation process in Morocco and the *embedded units* of the case were the *sociocultural, educational, and professional* systems targeted by the intervention [19].

### Study settings and participants

We conducted the study in settings affiliated to the MOH across two regions of Morocco involved in the implementation process. Using purposeful sampling, we selected stakeholders pertaining to the three systems under investigation. Participants were recruited in two stages: 1) we sent invitations to participate via email along with informational letters to potential key informants pertaining to the above mentioned systems using address lists provided by the training division of the MOH; posters were used to recruit midwifery students; and, 2) site visits were conducted by the first author in collaboration with the project coordinator to select key informants for focus groups. As such, key informants from educational institutes, health services, and governmental departments were approached in two regions: one located at the north of Morocco (Tétouan) and the second at Morocco's capital (Rabat). The reason for selecting those sites was that some activities of the AP were being actively implemented in the educational settings located in these regions. This would, consequently, allow for data collection from participants attending the training and the CBE program activities. Nevertheless, we were able to achieve variation on a range of views as midwifery educators attending the sessions came from a broad range of educational institutes (10) from across ten Moroccan regions. This process resulted in a total of two educational institutes, two health centers, two birthing homes, two maternity hospitals, the directorate of population, the training division, and two regional delegations of the MOH and the United Nations Population Fund’s (UNFPA) office taking part in the study. Our final sample consisted of 107 informants (106 key Moroccan informants and one international consultant). Table 1 presents characteristics of the participants.

**Table 1:** Characteristics of the participants in the study (*n* = 107)

Job title	N	Sex		Work experience Mean(range)/years^a^	Rural setting experience	Urban setting experience
					Yes	Yes
		Male n(%)	Femalen (%)		*N* = 83(%)	*N* = 83(%)
Midwife practitioner	17	0	17	11.29 (1–25)	16	17
Nurses	5	1	4	20 (9–28)	2	5
Obstetricians	6	5	1	7.66 (1–16)	3	6
Physicians	2	1	1	24.5	1	2
Medical directors	2	2	0	23.5	1	2
Senior nurse managers	2	1	1	16	2	2
Midwifery managers	2	0	2	26.5	2	2
Midwifery representatives	2	0	2	33.5	1	2
Midwifery educators	29	0	29	15.25 (6–32)	24	29
Midwifery students	15	0	15	N/A	N/A	N/A
Academic directors	2	1	1	32.5	1	2
Academic management staff	4	3	1	28.5 (24–37)	4	4
Medical administrative officers	7	6	1	19.85 (15–29)	7	7
Administrative nurse cadres	2	0	2	23.5	1	2
Health programmer	1	1	0	16^a^	1	1
Women	8	0	8	N/A	N/A	N/A
Consultant	1	0	1	29^a^	N/A	N/A
N	107	21(19.63 %)	86(80.37 %)		66(79.52 %)	100 %

^a^Work experience in years; *N/A* not applicable

### Data collection

We used qualitative methods to assess how well the AP was executed and to identify barriers and facilitators to implementation success. Data were collected through: focus groups (FG) (*n* = 20); individual interviews (*n* = 11) as well as from field notes, observation of educational sessions (30 h) and documents related to the implementation process (*n* = 16). Information about the quality of the implemented training activities was gathered post-hoc from session reports. By using a 1–5 point scale (ranging from very unsatisfied to very satisfied), satisfaction was assessed regarding five dimensions (objectives, content relevance, themes and presentations, acquisition of new knowledge about the midwifery role and practice in reproductive health care, organization of the training sessions).

Data was collected in June and July 2010, approximately 18 months after the AP’s launch. Selection of a range of key-informants was based on either their involvement in the activities’ implementation, or their role as health professionals or medical administrative officers who were exposed to the implementation efforts and were affected by the AP’s implementation. All interviews were conducted by the first author, a midwife, who was supported by a local assistant for participants who refused to be recorded. We used an interview guide, based on our conceptual framework "see Additional file 2". The questions covered the: level of information regarding the AP and the activities run; the activities’ level of implementation (e.g., the content of the ongoing activities, stakeholders’ involvement); and factors believed to be facilitating or hindering the implementation. The FG size varied from two to 10 members. We made sure groups were homogeneous to avoid mixing informants of different professional and organizational hierarchy. All interviews were conducted in French (only pregnant and postpartum women who received midwifery services were interviewed in Arabic).

The FG lasted 50 to 90 min, individual interviews ranged from 40 to 60 min. The subjects' verbalizations were audio-taped in one region while a few stakeholders refused to be recorded in the other region. Health system’s context, fear of being judged were among the main reasons for refusal. Detailed notes using abbreviations were written up for eight un-taped interviews which were reconstructed by the first author immediately after the interviews. Moreover, the researcher checked carefully the notes after the interview with each participant to ensure the accuracy of the recorded information. Direct observation of educational sessions took place at the validation midwifery’s program and train-the-trainers sessions, to examine the interaction between the participants, the coordinator, the consultant and the head of the training division and to assess the influence on the implementation process. All documents were requested from the consultant and the coordinator and were obtained except for the continuing education program’s report (Advances in Labor and Risk Management (ALARM)^3^ report) which was held by the Canadian instructors. Documents entailed e-mail correspondence between the international consultant, the coordinator and the training division (*n* = 10), project reports (*n* = 6) (UNFPA letters, session reports). As no other activities (e.g., profile, social marketing activities) were implemented, there were no data to be analyzed. Due to time constraints, analysis through an iterative process was not done; nevertheless, detailed notes were read throughout the data collection phase in order to gain a sense of the data prior to proceeding to further interviews.

### Data analysis

The analysis was conducted combining deductive and inductive approaches using QDA Miner software [20]. We used an initial coding list based on codes corresponding to the system’s four dimensions (*values, methods, actors, targets*) and the intervention characteristics suggested in our framework. We applied this coding scheme to the transcripts and conducted an inductive content analysis using constructivist thinking, allowing themes to emerge. Subsequently, clustered codes forming key themes were compared to CFIR constructs. The analysis proceeded through a longitudinal analysis of each corpus of data followed by a cross-sectional analysis of the entire data. Data reduction [20] consisted of: aggregating codes into key themes and assigning them to two categories (barriers, facilitators); mapping themes onto the CIFR’s constructs. In sum, the main themes were grouped under: intervention characteristics, values, methods, actors, targets of the three systems; and the constructs of the CFIR.

Concerning how well the AP was executed, data were organized according to the dimensions of the construct "Executing": i) fidelity of implementation (adherence to the planned activities; and coverage: whether the stakeholders who should be participating in the activities actually were) [21]; ii) intensity (quality) (satisfaction with the activities delivered); iii) timeliness of activity completion (extent to which tasks are completed in the planned timeframe); and, iv) degree of engagement (extent to which individuals are actively engaged in the implementation process). (See Table 2 for definition of codes).

**Table 2:** Codebook: definition of the codes

Mega-codes	Definition
Values	The values, beliefs, principles, laws and rules of the system
Methods	The organizational procedures used in the system and that include facilitators/barriers to implementation (e.g., communication and coordination mechanisms, availability and use of physical, human, financial resources, training, etc.)
Actors	The actors involved in the system’s functioning whose characteristics (e.g. capacities, motivation, attitudes) can facilitate/hinder the implementation process
Targets	The goals of the system which may facilitate/hinder the implementation process
Implementation process (Executing)	How well the implementation is carried out according to plan
Intervention	
Characteristics	Characteristics of the intervention perceived to influence the implementation (e.g., advantage, perceived difficulty of implementation)
CFIR’s constructs	Definition
Executing	Extent to which the activities of the plan were delivered as intended
Fidelity	Content adherence : adherence to the prescribed activities
	Coverage: whether the stakeholders who should be participating in the activities actually do
Intensity (quality)	The lived experience and the satisfaction towards the implemented activities
Timeliness of task completion	Adherence to timetable, compliance with deadlines
Degree of engagement	Active involvement of actors in the implementation process
External policy and incentives	The external strategies to spread interventions including: local, national policies, regulations, external mandates, guidelines and recommendations that influenced the decision to implement the intervention
Patient needs and resources	Extent to which patient needs are prioritized by the organization
Structural characteristics	Structural challenges (e.g., social architecture, centralization, maturity, size, etc.) that facilitate/hinder the implementation
Culture	Organization's beliefs, norms, values that affect the implementation
Networks and communications	The nature of social networks and of formal and informal communications within an organization
Readiness for implementation	Organizational commitment to its decision to implement an intervention
Available resources	Level of resources dedicated for implementation (e.g., money, training, education, physical space, equipment, etc.)
Access to knowledge and information	Access to information, knowledge and materials about the intervention
Characteristics of individuals/Personal attributes	Personal traits such as tolerance of ambiguity, motivation, intellectual ability, competence, capacity (e.g. leadership, involvement of leaders with the intervention to help make implementation successful
Intervention source	Perceptions of stakeholders about whether the intervention is externally or internally developed
Complexity	How complicated the intervention is in: duration, radicalness, number of steps required to implement
Relative advantage	Stakeholders’ perception of the advantage of implementing the intervention versus an alternative solution

### Scientific rigor

Data sources were triangulated by cross-checking the different point of views of stakeholders and by seeking several data sources in order to check for consistency [22]. A summary of the results was also sent back to eight participants for confirmatory purposes. Two co-authors other than the first author were involved in the codification and the matrix review to ensure accuracy of interpretations. One independent researcher provided additional input on the themes. Through discussion, consensus was achieved on the final themes. The findings were shared at the ICM Congress 2014 with a range of midwifery educators and practitioners, midwives representing the Midwives Association and the MOH, who had already participated in the study. This allowed us to obtain their feedback regarding recommendations to improve the implementation process.

### Ethical considerations

Ethics approval was obtained from the Research Ethics Committee of Université de Montréal, Québec, and the MOH in Morocco. Informed consent was obtained from all participants. Anonymity and data confidentiality were ensured.

## Results

Seven themes facilitating the implementation and 17 themes describing the barriers across the three systems and the intervention’s characteristics were drawn from the findings. Our results are consistent with the constructs of the CFIR domains (22 out of 24 key themes were captured by the CFIR constructs).

One notable finding is that 55 of all Moroccan informants (106) were not knowledgeable about the AP due to communication problems influenced to some extent by the structural characteristics of the Moroccan system. They were mainly health practitioners and managers (36/55) (e.g., midwives, nurses, obstetricians, medical directors) working in clinical settings. As access to information was deficient due to a lack of a formal communication strategy, we decided to inform the participants about the AP and the ongoing activities. We aimed, through instructive information, to raise awareness of implementation issues. Interviews were re-conducted and participants were asked to highlight factors that may help or constrain the implementation according to their knowledge of the Moroccan context. Furthermore, interviewees provided their perspectives on strategies to address barriers. As barriers and the potential solutions often mirrored one another, we decided to group both under the same themes.

### Executing the implementation

#### Fidelity

### Content adherence

The implementation analysis has shown a sizeable gap between planned versus delivered objectives. Emphasis has been placed on achieving only the first 3/9 objectives of the AP, related to: i) implementing the ALARM international program; ii) training the trainers on the competency-based approach (CBA); and, iii) developing and implementing a CBE program.

- The ALARM international program (objective1): Data analysis has shown that only three training workshops were held due to financial constraints. We were unable to assess the adherence to the planned activities and other dimensions due to lack of access to the workshop reports and to lack of well-defined activities related to this objective in the original AP.
- Train-the-trainers activities and developing and implementing a CBE program (objectives 2 and 3): Four sessions were held and focused on the reformulation of the program modules according to the CBA, and on training activities regarding the clinical (Humanization, sexual and reproductive rights, etc.) and academic concepts (Reflexive approach, etc.) required to prepare health professionals and educators to provide holistic care to the maternal-child population.

With regards to objective 2: Data analysis has shown that the training needs of the stakeholders were not identified by a committee as it was planned. They were delineated during the curriculum review sessions following the consultant’s request. Content of the training activities was delivered, however, according to what was planned regarding the clinical and academic concepts.

Concerning the CBE program (objective 3): Three sessions involving midwifery educators selected by the training division of the MOH led only to the elaboration of the referential for the CBE program and not to its implementation, and this was done mainly by the international consultant. Also, activities carried out drifted in some ways from what was intended. Data analysis revealed that a small committee had been set up including only six midwifery educators from one institute instead of creating a national committee bringing together a variety of midwifery educators from different institutes as originally planned. Moreover, it was dysfunctional due to lack of coordination between the training division, the consultant and the committee members which prevented them from carrying out their activities.

### Coverage

Data has shown poor coverage as regards to the intended stakeholders who were supposed to attend all the sessions set up for objectives 2 and 3. The selection process done by the training division targeted mainly midwifery educators instead of focusing on actors from the clinical settings and on medical administrative officers involved in policy-making. Restricted funds from UNFPA and the MOH resulted in a failure to include all actors in the process which was reported to compromise the future implementation of the CBA in clinical settings. Moreover, conditions for participation in the ongoing activities were unfavorable. Discontinuity as regards to participating to all implemented sessions was a concern expressed by midwifery educators. An attempt by the MOH to cover all institutions across Morocco has resulted in the selection of different participants at each session. Regular participation involving the same candidates was considered by many educators as crucial for maintaining informational continuity and successfully implementing the CBA’s activities. Covering the educational institutes and the clinical settings across the 16 Moroccan regions was stated by almost all participants as crucial to reduce potential resistance in clinical settings and enhance the future adoption of the CBA.

### Intensity (quality)

Our data covered the quality of the delivered educational sessions:

- The ALARM program: Many participants exposed to this program expressed their satisfaction regarding their positive experience with the training sessions. They found their training very instructive and practice-expanding. They learned to practice on anatomic models and gained a broader knowledge in dealing with obstetrical emergency cases. A medical administrative officer stated that the ALARM training provided participants with evidenced-based management of the causes of maternal mortality. Another academic director linked the training to the improvement of the midwives and physicians’ performance.
- Train-the-trainers activities: Participants reported to be very satisfied and satisfied with regards to the training received in terms of: objectives (16/18 participants), relevance of content (17/18), themes and presentations (16/18), acquiring new knowledge about the midwife’s role (17/18) and professional practice (16/18). Analysis of participants' responses showed they were rather satisfied with the interactive teaching methods used. Dissatisfaction was reported regarding the duration of the session (14/18) and the logistical organization (13/18). However, the content level was sometimes a source of difficulty (4/18) (e.g. critical thinking). Finally, the program’s presentation was frustrating given it intensiveness (6/18). Participants emphasized the need to be well prepared for implementing the CBA and to improve the logistical organization of the training sessions.

#### Timeliness of activity completion

The findings revealed a significant discrepancy between the actual time frame and the one outlined regarding the training session and the implemented program review activities. Reviewing the program’s sessions was postponed several times with a lag of several months between scheduled dates and those during which activities were carried out. Many reasons prevented the sessions from being organized on time such as authorizations from the MOH and administrative procedures related to the budget and to delays in allocating financial resources by UNFPA.

Moreover, many participants thought that the training division rushed the overall activities and scheduled them over short periods of time. The large amount of content to cover during a limited period of time (eight hour training sessions over six days) was cited by many participants as a major impediment to understanding. Sessions were held during a busy fall/spring period, making it difficult for midwifery educators to attend the program’s activities. Lengthening the training time sessions was recommended by many midwifery educators.

#### Engagement of key players in the implementation process

The extent to which participants are engaged in the different phases of the project was assessed through the perceptions of the informants and the document analysis. There was a noticeable lack of a team of individuals actively engaged and sharing the responsibilities for the implementation as it was originally designed. The process was organized in silos between the consultant, the head of the training division and the project coordinator. The consultant was the one leading the implementation of the educational activities, but following the directives of the training division. Thus, it diverged significantly from what was intended with regards to creating committees. Most notably, no activities were designed to engage actors in the process and to share information about the AP and even about the CBE program. Clearly, many participants were not even aware of it; and many others were considered as passive recipients experiencing delayed information about the ongoing activities, invited to execute the training division’s orders. Despite the consultant’s recommendations, no efforts were made to engage key stakeholders across the clinical settings specifically in order to counteract possible resistance in the future.

#### Perceived facilitators and barriers to implementation

Our findings have been categorized in two main categories*: facilitators* (7 themes) *and barriers* (17 themes) to the AP’s implementation.

Tables 3 and 4 show the complete list of themes, and representative quotations to illustrate facilitators and barriers according to the constructs of the CFIR in the three-systems; whereas Table 5 provides the characteristics of the AP acting as barriers and facilitators to the implementation according to the CFIR constructs.

**Table 3:** Facilitators to implementing the action plan in Morocco

	Mega codes	Sub-codes	Themes	Illustrative quotations
Socio-cultural System	Values	Values of the society	Primacy of human beings value and respect for human rights	*“…a facilitator is that the Moroccan society has changed its thinking over the past 10 years, it is more aware of everything that concerns rights and duties, it is aware of everything related to gender… for example concerning the women’s rights”*
	Methods	External policies and incentives	National and international policies to strengthen midwifery and to reduce maternal death	*“The action plan [the ongoing activities] stems from the national strategy, which facilitates a lot”*
	Actors	Attributes/Capacities	Strategic leadership to strengthen midwifery and to reduce maternal mortality	“*I think as regards to the support, the implementation is being taken seriously into consideration, at the strategic level, at the intermediate level and operational level. For the strategic level, this means at the Ministry of Health … this function can be attributed to the head of the training division who holds key position* ”
	Target	Ministry of Health and international agencies’ goals	Prioritizing maternal health in Morocco	*“I see that Morocco wants at all costs to follow the global directives… of the millennium goals”)*
		Patient needs and resources	Multidimensional demands for high quality services for the sake of women’s well-being	“*There is one environmental factor, it is the people, recipients who have become much more demanding and assertive towards the Ministry of Health, it is a facilitator because it incites the government, the Ministry to be vigilant and to move towards achieving what was stated in the national strategy regarding the reduction of maternal mortality”*
Educational System	Values	N/A		
	Methods	Readiness for implementation/Available resources	Intensified training support for midwifery teachers	“*She passed by groups to …, to adjust a vision or approach, and to provide guidance for going further in the process*”
	Actors	N/A		
	Targets	N/A		

*N/A* not applicable to data

**Table 4:** Barriers to implementing the action plan in Morocco

	Mega codes	Sub-codes	Themes	Illustrative quotations
Socio-cultural System	Values/Law/Rules	Structural characteristics	Bureaucratic top-down and centralized organizational structure in Morocco	*“The activities are centralized at the training division that deals with implementation, …,we look after the execution phase, I mean we only take orders from the top and receive information regarding the activities to execute”*
	Methods	Readiness for implementation/Available resources	Insufficient financial resources	“… *We have 16 areas that need to be covered, few people were able to attend the sessions, not all the staff, because of budget problem*”
	Actors	Capacities/Attributes	A truncated leadership to strengthen midwives	*“I think the other activities of the plan other than the training are not for the moment the priority of the policy makers, I mean they don’t have any interest or it is not yet official”*
		Attitudes	Attitudes of resistance of physicians towards midwives	*“Physicians are not going to … provide a backup mainly as regards to the activities intended to obtain a council board and change the law, and to have a clear statute”*
	Targets	N/A		
Educational System	Values	Culture/Philosophy of midwifery training and education	A dominant technocratic approach to midwives education and training	*“The training model is primarily based on a task approach, we are not prepared for the role and trained to perform a global practice, this principle of training could be a problem, a difficulty to put in place any further intervention”*
	Methods	Readiness for implementation/Available resources	Limited availability of human, physical and technical resources for implementing the training activities	*“Computers made available to participants were not very helpful, without any protection against virus and software to download documents was not available. Thus, they [midwives] had great difficulty to have access to some references … and make presentation in plenary sessions”*
			Deficient educational support for midwifery teachers	*“As I have mentioned a while ago, the lack of material resources …, the lack of human skills to support this change, we do not have till date competent midwives who are trained according to the competency-based approach to train the future midwives”*
			Deficient access to information regarding the action plan	*“In general, when there is an action plan, few people are aware of the entire plan, it remains where it was developed at the top of the chain at the policy level …. I think, it’s the system here, it does not provide any information…”*
		Networks and communications	Inadequate mechanisms of coordination and collaboration within and across the educational institutes	*“We are all victims of the lack of coordination between our leaders and the educational institutes, we have done a crazy work during the first year …in September-October 2009, we asked for a feed-back, and we suggested a deadline for receiving feedback, comments were not submitted to the consultant, we even called for a meeting to get feedback, we have not received a response due to the issue of coordination”*
	Actors	Attributes/Capacities	Difficulties in adapting teachers skills to the competency-based approach’s needs	*“If we teachers found it difficult… to understand, what would it be for students who fail in the first year to speak French, are not able to reason properly, students will not be able to understand the new concepts, they will face difficulties in assimilating the various "savoir, savoir-faire"*
			Attributes of students unfavorable to educational change	
	Targets	N/A		
Professional System	Values	Culture	Workplace culture resistant to innovations	*“The change is more difficult, it takes longer time, people's mentality is hard to change, they are not open to a new model of care primarily the medical community”*
	Methods	Readiness for implementation/Professional support	Lethargy of the midwifery association towards the profession	*“Their presence [Professional Association] is symbolic, we do not have a well-developed association, we are not part of it, in my opinion it no longer exists, no activities are done to strengthen the role of the midwife. At Rabat, there are no activities to strengthen the midwife’s role”*
			Deficient access to information as regards to the Action Plan	*“Here, at the maternity hospital, we did not receive any information from the Ministry of Health regarding the plan, and the ongoing training activities at the educational institutes”*
	Actors	Attributes	Lack of motivation of practicing midwives to embrace change	*“We are not motivated, so how do you want to set up a plan, we are like a simple tool for doing tasks, even for planning, for making decisions, for implementing a project, we are not considered nor consulted”*
	Targets	N/A		

*N/A* not applicable to data

**Table 5:** Facilitators/Barriers related to the characteristics of the action plan

	Megacodes	Themes	Illustrative quotations
Facilitators	Relative advantage	Multilevel perceived benefits of the action plan	*“Midwives are going to be trained to be autonomous in order to train the society to be autonomous and so to transfer this competency to young girls, to women, children and men*”
Barriers	Complexity	Complex nature of the action plan	*“A lot of guidance and support, many steps technically, organizationally, and lots of patience because it is complex and we are speaking here about many stages”*
	Intervention source	External source of the plan	*“The Ministry of Health is concerned with reviewing the program, despite that I [The consultant] developed a global plan”*

Over all, the results show that barriers generally outnumbered the facilitators. In general, the barriers covered the three systems and the intervention whereas the facilitators belonged mainly to the sociocultural system which was found to have a great influence on the implementation process as a whole.

## Discussion

Our results showed that implementing the AP is a complex process that is influenced by a variety of interacting factors pertaining to the three systems as well as to the intervention itself. The study offers insight into the actual factors affecting the ongoing educational activities; and the potential barriers to adopting and implementing the other activities, that were on standby, in the future. Our findings are relevant to the adoption and implementation stages of the change process [23] since factors were reported by actual and potential users who were not exposed to the AP. Taking into account these factors across these stages is considered as relevant for tailoring implementation strategies and adapting the intervention to specific contexts [12, 24, 25].

### The extent of implementation

We have found poor fidelity, deficiency in the timelines for the completion of activities, and lack of actors’ active engagement in the process. Funding constraints and the bureaucratic and centralized organizational structure in Morocco were challenges to executing the AP and have played an important role in the reported failure. Despite that the AP was welcomed during the validation phase of the project, mid-level policymakers attempted to implement only the educational activities, giving the impression that a final decision on fully adopting the AP has not been made. Referring to Rogers [26], the “Knowledge, Attitudes, Practice” gap results when decision-making leads to active rejection for adopting the innovation "in toto" (p.177). In our study, this gap stemmed from authority innovation-decisions [26] where individuals in the various Moroccan systems have no influence in the innovation-decision. The AP’s drift is mainly the result of barriers present in the sociocultural system which in turn impacted the educational and professional systems.

One of the important implementation gaps that emerges is the lack of professionals’ active engagement in the implementation process. Consistent with our findings, many studies have highlighted that effective implementation strategies are those that achieve an active involvement of front-line professionals in the change process [27–29]; this has many implications such as increasing knowledge about the innovation, gaining a sense of ownership [9], thus, decreasing the potential for resistant attitudes and, also, creating a greater commitment on the part of the participants [26, 30, 31].

### The facilitators and barriers encountered with respect to implementation

#### The three-system level

We emphasize the interactions that occur mainly between a range of dominant factors across the three-systems and at the intervention’s level illustrating how our framework is essential for understanding the implementation process. The three systems work in dynamic interaction that challenge the AP’s adoption and implementation while recognizing the fundamental role that the sociocultural system plays in the implementation process. Misalignment of the *values, methods, actors and targets* of the sociocultural system with the *values, methods and actors* of educational and professional systems on one hand; and with the intervention, on the other hand, are likely to be the greatest impediments to the adoption and full implementation of the AP. Our study revealed key facilitators in the sociocultural system essentially related to the values prevailing in the Moroccan system (e.g., respect for human rights) that mainly drove the national and international policies to reinforce midwifery, the presence of leaders (the head of the training division) who strongly advocated for the implementation of the educational activities. Nonetheless, many barriers in the sociocultural system have counteracted the facilitators’ effects and have also affected the other systems. Consequently, a lack of alignment between, on one hand the *values* promoted (e.g., primacy of human beings) and the high priority given to maternal health; and, on the other hand, the *methods* set up (e.g., insufficient financial resources), capacities (e.g., truncated leadership) and attitudes of *actors* (oppressive attitudes of doctors) to reach this target, was evident. Three important barriers in the sociocultural system found in the system’s structure (e.g., centralized structure), system’s methods (e.g., insufficient financial resources) and the actors’ levels (e.g., truncated leadership to reinforce midwives) exerted a coercive influence on the educational and professional systems. For example, the hierarchical bureaucratic structure in Morocco, characterized by a high degree of centralized lines of control, and complex administrative procedures was a big hindrance to implementing any activity. Our findings are in agreement with Macfarlane et al. [32] and Edward III [33], who have also identified the bureaucratic top-down structure as barriers to effective implementation of human resources strategies and public policy. Scott and Caress [34] suggest to move away from the rigid top-down approach and to involve staff in decision-making processes in order to increase ownership.

This structure has contributed to the limited access to knowledge about the AP’s activities among many stakeholders in the three systems. Our results do echo previous findings that emphasized the necessity of effective communication in managing a healthy change process and its benefits in overcoming resistance [28]. Effectively, promoting an understanding of the innovative efforts, and enhancing ownership of the innovation were among the benefits reported [35].

Under the *Actors* dimension, the negative attitudes of the medical group towards the future expansion of midwives’ competencies should be taken into consideration as it has been highlighted in many studies [36, 37].

With regards to how the sociocultural system affected the two other systems, insufficient budget allocated by the MOH and UNFPA played an influential role on the readiness of the educational system in terms of lack of adequate resources to implement the educational activities intended. The data suggest that sustained financial support is also a major concern. Providing a receptive context and devoting sufficient resources to support effective implementation of work role redesign interventions are considered as key elements [8] to bring the AP to fruition and to produce the intended results [38–40]. Moreover, financial constraints stood out as a barrier to mobilize the midwifery professional association. The spread of an innovation often rests on professional organizations which are identified as taking the lead to activate this process [41]. Nevertheless, our findings showed the absence of revitalization of the midwifery Moroccan association which does not seem involved in promoting the profession.

Many barriers were also identified as constraining the adoption of the forthcoming change at the *actors*, *values* and *methods* levels in the educational and professional systems. Characteristics of the actors (e.g., educators’ skill deficits, lack of familiarity with the CBA’s needs) and the unmotivated midwifery workforce would affect their receptiveness to change. Concerning the values level, the prevailing biomedical culture in the workplace and the technocratic philosophy of midwifery education were perceived as incompatible with the forthcoming holistic approach and as hindering the successful implementation of the CBA. This is in line with earlier studies that found that organizational culture deeply embedded in traditional organizational forms is highly resistant to the introduction of role redesign interventions [32, 40].

Moreover, coordination and collaboration mechanisms and lack of collaborative teamwork were perceived as barriers to the future implementation of the CBA. Tensions between the norms of midwifery practice and biomedical obstetric care underpinning the coordination challenges identified in the Moroccan context were similar to those found in Colvin et al.’s review of midwifery task-shifting programs [7].

### Intervention characteristics

There was a general consensus concerning the AP’s (and the CBE program) potential to improve quality and continuity of care, and to clarify the midwifery role. Colvin et al. [7] found that midwifery up-skilling often entailed the same advantages. Future adopters’ positive perception of the anticipated benefits of the AP might contribute to make it visible to mid-level policymakers and to be a push factor to reverse the decision of rejecting the entire AP and reconsider its adoption [26, 39].

However, the intervention source and the complexity of the AP perceived as being challenges to its implementation should be taken into consideration. The AP designed by the consultant did not align with the middle-level policy makers’ initial mandate (strengthening the midwives’ education). Differences in priorities between both parties and the centralized structure in the Moroccan system have contributed to obstructing the adoption decision of the global AP. It is suggested [38, 42] that in order to adopt a change, it must resonate with the current perceived needs and belief systems. Complexity of the AP, the radical change needed on many levels confirm earlier research in this area showing that innovations broad in scope may be difficult to implement [18, 38].

Interestingly, we observed significant alignment between the various activities not taken into consideration by the mid-level policymakers, and the contextual needs called forth by the front-line providers (e.g., involving potential users in the change process, improving readiness). These results emphasize the importance for policymakers to pay attention to the key stakeholders’ needs to put in place concomitant change process at the sociocultural, and professional system levels in order to address potential adopters’ interests and, ultimately, meet health services’ needs. Authoritative decisions have been reported to reduce the chance of successfully implementing an intervention [12, 38]. We predict that the successful implementation of this intervention in the future lies in the extent to which policymakers are willing to shift from a narrow focused model of change to a focus on the whole system. Ignoring the systemic nature of the change process will not result in the desired outcomes.

### Recommendations

Our findings offer important implications for policymakers, health care planners, and researchers, to redirect future implementation efforts. They suggest important lessons for tailoring implementation strategies to the barriers [12] identified in the Moroccan context. We recommend the following:

- Consider using further knowledge transfer strategies (e.g. interactive workshops) in addition to the brief reports that have already been sent to the MOH secretary office coordinator. This will help explain, to policy makers and key stakeholders, the conceptual foundations for changing a health professional role, to persuade them about the relevance of the suggested intervention and motivate the implementation of the entire AP. Implementation consultants need to work in partnership with policymakers to redesign the AP’s activities and implementation strategies.
- Adopt a collaborative approach in the implementation efforts as a substitute for a top-down approach to facilitate the implementation process. An effective strategy would be to actively engage a broad range of key stakeholders pertaining to the three systems in the different steps of the change process (redesigning the AP’s implementation strategy, executing, evaluating the change). Involving potential users in the innovation process has been considered a "*condition sine qua non*" based on several implementation theories [12] to promote committed use of the intervention [25]. Allocating responsibilities to a team of individuals (e.g., obstetricians, midwives, educators) would help building a strong coalition of stakeholders with complementary expertise to support the implementation process [42]. This would help create, in the future, a receptive culture in clinical and educational settings, and prevent professional culture clashes. Champion teams have, in fact, been considered as more efficient and effective than lone champions [29].
- Enhance communication mechanisms by establishing effective dissemination and information strategies (e.g., workshops, brochures) supporting open lines of communications among the stakeholders involved in the process.
- Improve organizational readiness (e.g., increase financial, human and material resources to implement the AP, provide more relevant training sessions).
- Adopt a participatory approach in the future by involving relevant decision makers and key stakeholders as research partners in the evaluative process. This will promote collaboration with participants and help feelings of ownership over the findings [22]. Obtaining commitment from stakeholders to monitor the implementation’s progress through regular feedback will contribute to the adoption of strategies to continuously improve implementation efforts and to promote shared learning [18, 22].

### Strengths and limitations

We present information based on real-life experiences and representing heterogeneous key stakeholders’ views pertaining to three systems which help increase the validity of our findings. Another strength of this study is the use of a conceptual framework adopting a holistic approach for analyzing the implementation of a health professional role change.

Some limitations should be noted. Due to limited financial resources, information was gathered during a two month period. An ongoing assessment is thought to be better than a punctual implementation evaluation, nevertheless a documentary analysis and triangulation of data helped track the implementation process over time. Also, the factors mentioned by respondents who were not exposed to the AP, address the adoption rather than the implementation stages. A lack of well-defined activities related to the first objective of the AP and of access to ALARM reports did not allow us to assess how well this objective was executed with regards to fidelity and timeliness. Also, data regarding the quality dimension were extracted from secondary sources of information and gathered from interviews. Incorporating quantitative and qualitative measures to assess each objective may provide a better understanding of the extent of implementation. Finally, as a case study method is adopted, our results may not be generalizable across all contexts; however, they may help the reader decide to what extent they are transferable to similar settings.

## Conclusions

Our findings are novel within the field of implementation research regarding interventions aiming to strengthen the midwife’s role. Policymakers should pay particular attention to the influential factors in order to rectify the AP’s shortcomings and to improve implementation. Further research must be undertaken to better understand why the implementation of the overall AP did not pass the decision-making agenda. Our results have potential implications for other countries attempting to implement interventions to strengthen the midwife’s role and to reduce maternal mortality.

## Additional files

Additional file 1:
**The action plan.** (DOCX 17 kb) Additional file 2:
**Interview guide.** (DOCX 23 kb)

## Abbreviations

AP: Action Plan
ALARM: Advances in Labour and Risk Management
CBA: Competency-Based Approach
CBE: Competency Based Education
CFIR: Consolidated Framework for Implementation Research
FG: Focus group
MDG: Millennium Development Goals
MM: Maternal Mortality
MOH: Ministry of Health
UNFPA: United Nations Population Fund

## Footnotes

1 The *sociocultural system* represents the larger societal system, encompassing political (governmental bodies), and social systems at large (civil society). It concerns the values of society laws and regulations. The *educational-system* represents the educational principles and methods for optimizing the preparation of health professionals to attain the training goals. The *professional system* corresponds to the characteristics pertaining to the profession. It outlines the values of its members, practical approaches used; also the organization of the profession including its relationships with other professional groups; and the goals of developing their role.

2 Systems approach: It is defined as « a general conceptual orientation concerned with the interrelationships between parts and their relationships to a functioning whole, often understood within the context of an even greater whole » (Trochim, Cabrera, Milstein, Gallagher, & Leischow, 2006, p. 539).

3 The ALARM international program: is a continuing education program for health professionals responsible for the delivery of emergency obstetric and newborn care that addresses the causes of maternal mortality, the sexual and reproductive rights approach (Lalonde, Beaudoin, Smith, Plourde, & Perron, 2006).
