# Supplementary material for: Analyzing barriers and facilitators to the implementation of an action plan to strengthen the midwifery professional role: a Moroccan case study
Source: BMC Health Serv Res. 2015 Sep 15;15:382. doi: 10.1186/s12913-015-1037-3 (PMC4571078; doi:10.1186/s12913-015-1037-3)
Supplement: Additional file 1: — The action plan. (DOCX 17 kb) [file 12913_2015_1037_MOESM1_ESM.docx]

**Additional file 1:** The action plan

| **Components** | **Objectives** | **Interventions** |
| --- | --- | --- |
| **Educational** | ***Objective 1:*** Put in place a continuing education program (ALARM program) | Planning the ALARM international program  Provide training for all trainers in Morocco (3 -4 workshops)  Provide training for all trainers in Morocco (3- 4 workshops) with the Moroccan instructors |
|  | ***Objective 2 :*** Organize and put in place a training program for the trainers | Create a committee to identify the training needs  Identify persons and institutions who have to participate and invite them to take part of the committee (educators, practicing midwives and members of the multidisciplinary team, health administrators)  Provide training on clinical and academic concepts of the competency-based approach |
|  | ***Objective 3 :*** Develop and put in place a CBE program | Create a national committee gathering educators from all educational institutes across Morocco  Organize meetings of the national committee and set timelines for the various activities  Follow the stages of the Guilbert framework for developing and implementing the CBE program |
| **Professional** | ***Objective 4 :*** Create and mobilize the midwifery committee profile | Identify persons and institutions who have to participate and invite them to take part of the committee  Organize meetings of the committee and set timelines for the various activities. |
|  | ***Objective 5 :*** Revitalize the central and local associations of Moroccan midwives | Encourage the central and local associations to meet together  Mobilize and intervene on the various negotiation tables related to the reproductive health care and to the profession  Reinforce the identity of the midwife and her professional recognition |
|  | ***Objective 6 :*** Mobilize the institutional and interdisciplinary lobbying | - Get the different members of the multidisciplinary team in reproductive health to meet and discuss their collaboration - Get the institutional representatives to meet with the representatives of the midwifery profession to discuss the midwife’s role and how to restructure her practice |
| **Socio-political, legal** | ***Objective 7 :*** Set up the marketing activities for the profession | Create a task force for marketing  Organize various activities for enhancing the midwifery role in society |
|  | ***Objective 8*** : Create and mobilize the committee on regulation and law, and governmental lobbying | Identify persons and institutions who have to participate and invite them to take part of the committee  Organize meetings of the committee and set timelines for the various activities |
|  | ***Objective 9** *:*** Ensure the mobilization of human resources planners and the assignment of human and material resources in the Moroccan system | Negotiate with the leaders in charge of the distribution of human and material resources in the health care system: members of the committees of the midwifery profile and lobbying. |
| **Total** | **9 Objectives** |  |

*Objective 9: was pursued through the national strategy 2008-2012 for accelerating the reduction of maternal and infant mortality
