# Supplementary material for: Analyzing barriers and facilitators to the implementation of an action plan to strengthen the midwifery professional role: a Moroccan case study
Source: BMC Health Serv Res. 2015 Sep 15;15:382. doi: 10.1186/s12913-015-1037-3 (PMC4571078; doi:10.1186/s12913-015-1037-3)
Supplement: Additional file 2: — Interview guide. (DOCX 23 kb) [file 12913_2015_1037_MOESM2_ESM.docx]

**Additional file 2**: Interview guide

Interview ID:……….

Name of:

- Country:………
- Region, province:.........

Area (city):……….

Name of the:

- Setting where the interview took place:……….

Interview:

- Date:…….…
- Time:……….

Interview recorded:

🞎 yes (Interview Record ID)……….

🞎 yes (Interview Record ID), partially (*explain why*)……….

🞎 no (*explain why*)……….

Language interview:….......

**Introduction:**

As part of a national strategy for reaching the Millennium Development Goals 4 and 5 in Morocco – to reduce maternal and infant mortality - an action plan was developed to strengthen the midwives’ professional role in collaboration with the Ministry of Health, UNFPA’s office in Morocco. The purpose of this study is to assess the level of implementation of the action plan and to understand barriers and facilitators affecting the adoption and implementation of the action plan that may prevent reaching the targeted outcomes. The objective is to improve implementation of the current intervention. We want to talk to you about your experience with the action plan. This will help us to understand the challenges of adopting and implementing the action plan and its various activities aiming to strengthen the midwife’s role in Morocco. By sharing your experience, we hope to learn how this action plan could be improved in the future.

I am going to ask you a series of open-ended questions, and to record the interview. We will make sure that data will be kept confidential. Data files will be kept in a secure place and will be held on computer protected by password. Please do not hesitate to share your experience and thoughts related to any of the mentioned topics.

**Information about interviewees:**

Number of participants:……….

- Name:……….
- Sex:………….
- Profession:……….
- Position:………….

Professional experience:………. (in years)

Urban area:………...

Rural area:…………

- Pratice setting (Type, location):……….

**Implementation Experiences:**

1. **Level of knowledge :**

Are you informed about the action plan that has been developed and about its various activities that have been implemented or are being implemented to strengthen the midwifery professional role in Morocco? (How? By whom?)

**2. Extent of implementation (Executing):**

Can you tell me about the action plan? And about its activities? Have the activities related to the different objectives been implemented according to what was planned?

(if yes) Can you describe this?

(if not) Why not?

- Which team is involved in the process? What are their roles? What about your involvement? In which activities you were /are involved?
- Have you participated in any activity of this plan? If so, which one?
- Describe to me the activity, its content, how it unfolded /or is unfolding, your experience.

**3.Factors facilitating and or hindering the implementation:**

**3.1 *Characteristics of the action plan:***

According to you, are there some characteristics related to the action plan and its activities that can be considered as barriers or facilitators to its implementation?
Probe: In respect of the following:

- The advantages and benefits that the action plan has
- How complicated is the action plan?
- The resources used to implement

You can express yourself on any other characteristic that seems facilitating or constraining.

**3.2 *Educational, professional and socio-cultural systems:***

3.2.1 Now, I would like to ask you about the factors that can be considered as facilitators and or barriers to implement the activities of the action plan:

At the level of the following dimensions:

- Methods (probe : interprofessionnal relationships, ressources, communication, etc.)
- Values (probe: rules, beliefs, law, regulation, culture)
- Actors (probe: motivation, capacities, attitudes)
- Targets

At the :

- Educational institutes ?
- Clinical settings (e.g. maternity, health centers)?
- Professionnal associations (midwives)?
- Various committees of the action plan?

3.2.2 How do you think the Moroccan government, the Ministry of Health and the different directorates in Morocco, the community, the international agencies did contribute or are contributing to the implementation process?

How do you think these levels have supported and or hindered the implementation of the activities?

**4. In your opinion, to date, what were (are) the major barriers and facilitators to implementing the action plan and its various activities?**
